# Therapeutic cancer vaccines in pancreatic cancer

**DOI:** 10.3389/fimmu.2025.1674743

**Published:** 2025-10-20

**Authors:** YuHan Wang, Peng Huang, Chun Li, ShengJin Tu, Hua Yang

**Affiliations:** ^1^ Department of General Surgery, Zigong Fourth People's Hospital, Zigong, Sichuan, China; ^2^ Division of Abdominal Tumor Multimodality Treatment, Cancer Center, West China Hospital, Sichuan University, Chengdu, Sichuan, China

**Keywords:** pancreatic cancer, vaccine, immunotherapy, tumor antigen, clinical application

## Abstract

Pancreatic cancer is a leading cause of cancer-related mortality, and unfortunately, the prognosis for this stubborn tumor type has remained bleak for an extended period. Current treatment options are limited and often ineffective, highlighting the need for innovative therapeutic strategies. Research in tumor immunotherapy has advanced significantly over the past few decades, leading to numerous studies progressing to clinical evaluation. Cancer vaccines are regarded as a promising therapeutic approach for treating pancreatic cancers. Cancer vaccines offer distinct advantages, especially for patients who are unresponsive to other treatments, as they can elicit broad and long-lasting T cell responses. This review offers a thorough examination of recent preclinical and clinical results of therapeutic cancer vaccines in pancreatic cancer therapy, highlighting the recent advancements in vaccine delivery platforms. Despite ongoing clinical efforts to develop cancer vaccines, there has not been a breakthrough in clinical outcomes, primarily due to substantial challenges including the low mutation burdens, the assessment of immune responses in preclinical models, and the intrinsic resistances to cancer vaccines. By tackling these challenges, cancer vaccines can expand the possibilities of personalized immunotherapy for pancreatic cancer.

## 1 Introduction

Pancreatic cancer is typically a fatal disease characterized by resistant and chronic nature (1). According to the global cancer statistics, there were 511,000 new cases of pancreatic cancer and approximately 467,000 deaths, making it the sixth leading cause of cancer-related mortality worldwide (2). The incidence and mortality rates of pancreatic cancer are influenced by various risk factors, including long-term smoking, obesity, chronic pancreatitis, and concurrent diabetes (3). Pancreatic ductal adenocarcinoma (PDAC) is the main type of pancreatic cancer, and approximately 50-60% of PDAC patients present with distant metastases, and 5-year survival rate is approximately 13%, which is attributed to its early systemic dissemination and aggressive local growth (4). The current treatment strategy for advanced pancreatic cancer consists of standard chemotherapy; however, it only presents a minor survival advantage due to the desmoplastic and chemo-resistant features (5, 6).

Immunotherapy leverages the patient’s immune system to target and destroy tumor cells, leading to a transformative effect on the treatment of human cancers (7). Immunomodulators, immune checkpoint inhibitor (ICI), and adoptive cell therapy provide promising strategies for solider tumors, including melanoma, lung cancer, colorectal cancer, and liver cancer (8–11). Early-phase trials assessing single-agent CTLA-4 or PD-1 blockade in PDAC showed little clinical activity, with no objective responses observed (12). Adoptive cell therapy (ACT) represents a novel type of immunotherapy that involves isolating a patient’s immune cells and genetically engineering them to mount tumor−specific responses (13). In pancreatic cancer, active investigations include chimeric antigen receptor T cells (CAR−T), CAR−NK cells, tumor−infiltrating lymphocytes (TILs), T−cell receptor (TCR)–engineered T cells, and cytokine−induced killer (CIK) cells. However, translation to solid tumors, particularly pancreatic cancer, remains early, and clinical outcomes to date have been limited (13–15). These unfavorable results are likely driven by PDAC’s profoundly immunosuppressive tumor microenvironment and intrinsically poor immunogenicity that limit the efficacy of immunotherapies (16, 17). Therefore, there is growing interest in investigating innovative immunotherapeutic strategies, such as therapeutic cancer vaccines, to tackle these challenges posed by the hostile TME. The objective of therapeutic cancer vaccines is to stimulate the generation of effector T cells, leading to the establishment of a lasting immune response against specific tumor antigens and eradicating cancerous cells (18, 19). In contrast to conventional monoclonal antibodies and small molecule inhibitors, cancer vaccines provide various benefits, such as reduced nonspecific effects, a wide therapeutic index, and the durable immunological memory (20, 21). Additionally, cancer vaccines enable precise targeting tailored to the specific traits of individual tumors (22, 23). As a result, vaccination represents a promising strategy for personalized treatment of pancreatic cancer, addressing the complexities introduced by tumor heterogeneity.

In this review, we offer an extensive overview of the current landscape of therapeutic cancer vaccines and their clinical applications in pancreatic cancer. Furthermore, we discuss the resistance mechanisms that tumors present and assess the potential benefits of combination therapies, which may help to enhance the effectiveness of cancer vaccines in the treatment of pancreatic cancer. Moreover, we outline their advantages, limitations, and challenges posed by vaccine technology and provide future perspectives on developing therapeutic vaccine in pancreatic cancer.

## 2 Clinical landscape of cancer vaccine in pancreatic cancer immunotherapy

**Figure 1:**
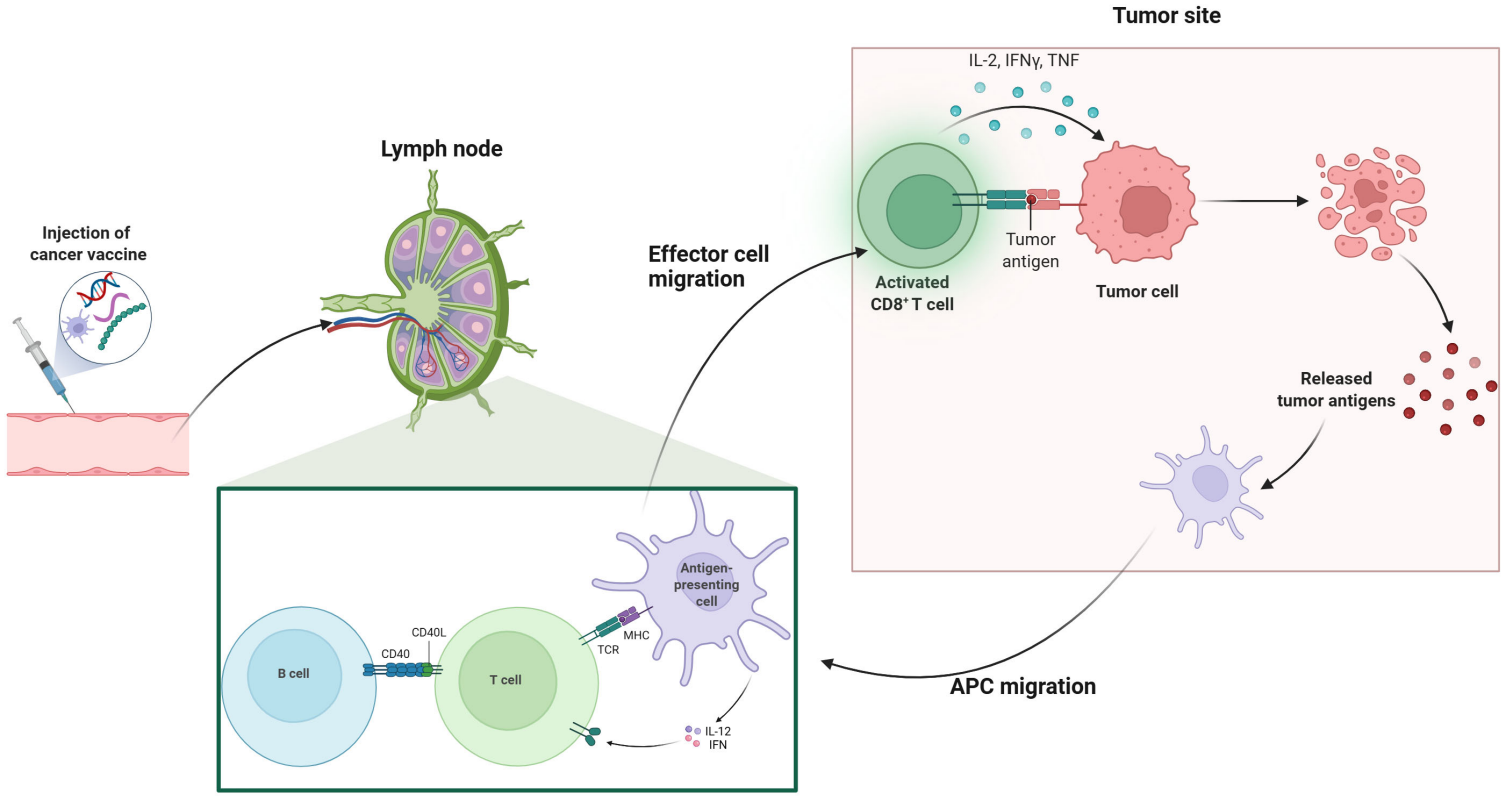
Mechanism of cancer vaccines. This illustration depicts the process of cancer vaccination and subsequent immune response activation. The series begins with the injection of a cancer vaccine, which then leads to the activation of T cells in the lymph nodes. Effector T cells migrate to the tumor site, where they recognize and target tumor antigens on cancer cells. Activated CD8+ T cells release cytokines such as IL-2, IFN-γ, and TNF, which enhance the immune response. Antigen-presenting cells (APCs) also migrate to the tumor site, facilitating further activation of B cells and T cells through interactions with CD40 and MHC-TCR complexes. This coordinated immune response aims to eliminate tumor cells and promote the release of additional tumor antigens, potentially enhancing the overall effectiveness of cancer immunotherapy.

After vaccination, innate immune cells such as natural killer (NK) cells, neutrophils, and macrophages rapidly identify foreign substances through pattern recognition receptors (PRRs), initiating specific immune responses (24). Distinct dendritic cells (DCs) subsets differentially prime defined T-cell lineages. With maturation, DCs traffic to secondary lymphoid tissues, particularly the draining lymph nodes (25). DCs capture and process peptides in peripheral tissues and present them via major histocompatibility complex (MHC) molecules. Endogenous proteins activate CD8+ T cells, while exogenous proteins are presented to CD4^+^ T cells as antigen-MHC-II complexes (26). APC-mediated activation of CD8^+^ cytotoxic T lymphocytes (CTLs) exit lymphoid organs, infiltrate tumors, and exert anti-tumor effects by inducing apoptosis in tumor cells through granule exocytosis (involving perforin and granzymes) and engagement of death receptors (such as Fas ligand and tumor necrosis factor-related apoptosis-inducing ligand) (27, 28). Therefore, the intricate interactions among immune components significantly influence the efficacy of cancer vaccines (**Figure 1**). Established delivery platforms for vaccines include, DC, tumor cell, nucleic acid and peptide vaccines, while emerging technologies are also under investigation (29, 30). In this section, we summarize current landscape of cancer vaccines in pancreatic cancer (**Table 1**).

**Table 1:** Selected clinical trials of cancer vaccines in pancreatic cancer.

Clinical trial number (Phase)	Antigen	Type	Combined therapies	Responses	Adverse events	Reference
UMIN000004855(phase I )	WT1	DC	gemcitabine	The DCR and mOS were 60% and 243 days	well-tolerated	(31)
Phase I/II	WT1	DC	gemcitabine	mPFS and mOSwere 4.9 and 9.6 months, respectively	well-tolerated	(32)
Phase I	WT1	DC	S-1	2-year OS rate was 62.5%	well-tolerated	(33)
Phase I	WT1	DC	chemotherapy, radiation	mOS and mPFS were 1796.5 and 607 days, respectively	well-tolerated	(34)
jRCTc030190195(phase I)	WT1	DC	nab-paclitaxel plus gemcitabine	Seven patients achieved a partial response.	grade 1 skin reactions	(35)
phase I/II	MUC1	DC	/	mOS was 26 months for all patients.	well-tolerated	(36)
Phase I/IIa	WT1/MUC1	DC	/	OS and RFS at 3-years from the time of surgical resection were 77.8% and 35.0%,	well-tolerated	(37)
NCT00569387 (Phase II)	algenpantucel-L	WTC	Gemcitabine, 5-fluorouracil and radiotherapy	12-month DFS was 62 %, and the 12-month OS was 86 %	injection site pain and induration.	(38)
NCT01836432 (Phase III)	algenpantucel-L	WTC	FOLFIRINOX or gemcitabine/nab-paclitaxel)	No survival benefit	well-tolerated	(39)
Phase Ib	GVAX	WTC	ipilimumab	mOS (3.6 vs. 5.7months)	20% of patients had grade 3/4 adverse events	(40)
NCT0245198(Phase II)	GVAX	WTC	Nivolumab and urelumab	Improved DFS and OS	well-tolerated	(41)
NCT04161755 (Phase I)	RO7198457	mRNA	Atezolizumab and mFOLFIRINOX	The median OS and RFS of the patients in the safety-evaluable cohort were not reached	well-tolerated	(42)
NCT02854072 (Phase III)	GV1001	Peptide	gemcitabine/capecitabine	Improved mOS (11.3 vs. 7.5 months) and TTP (7.3 vs. 4.5 months) compared to the control group	Grade >3 adverse events were reported in 77.3% in the GV1001 group	(43)

### 2.1 Cell-based vaccine

Cell-based cancer vaccines utilize the patient’s own immune cells, particularly DCs, to process both soluble and particulate antigens (44). Additionally, tumor cells are recognized as valuable antigen sources for vaccines, presented in multiple formats, including whole tumor cells (WTC) and tumor cell lysates (45). Both DC and WTC-based vaccines are evaluated in the clinical trials of pancreatic cancer. DCs play a crucial role in the immune system by effectively presenting both exogenous and endogenous antigens to T cells, thereby triggering adoptive immune responses (46). By showcasing tumor-specific antigens, DCs can trigger the generation of cytotoxic T lymphocytes that specifically recognize and destroy cancer cells (47). DCs are specialized APCs that are highly effective in generating robust immune responses and maintaining tolerance to self or benign foreign antigens, making DCs an appealing tool for developing immunotherapeutic strategies (48). Since the study has assessed the efficacy of DCs pulsed with melanoma-associated antigen (MAGE) 1 for treating melanoma patients, emerging evidence has demonstrated treatments based on DCs are safe, even in patients with advanced cancer (49). Additionally, DC-based therapies can enhance immune responses that lead to sustained remissions, providing optimism for enduring outcomes (50). The Wilms’ tumor 1 (WT1) antigen has been recognized as a highly effective target in various cancer types, including pancreatic cancer (51). WT1-peptide pulsed dendritic cell (WT1-DC) vaccine combined with gemcitabine chemotherapy resulted in durable specific T cell immune responses, which were associated with significant improvements in survival in advanced PDAC patients (31–35). Further studies broadened the insights into the potential clinical application of WT1-DC vaccines as evidenced by vaccine injection combined with chemotherapy exhibited safety and effectiveness in resected PDAC patients (52). And WT1-DC vaccine in conjunction with multimodal treatments including chemotherapy, irradiation and surgery was related with longer overall survival time (53). Moreover, the chemoimmunotherapy regimen consisted of nab-paclitaxel plus gemcitabine combined with WT1-DC vaccination regulated TME and facilitated conversion surgery for advanced pancreatic cancer patients (54). A chemoimmunotherapy approach combining a WT1−DC vaccine with multi−agent chemotherapy was observed to reprogram the TME toward an immunostimulatory phenotype, permitting conversion surgery in 7 of 9 patients with unresectable pancreatic ductal adenocarcinoma (55). Although early observations are encouraging and informative, definitive clarification of the treatment effect attributable to WT1−targeted chemoimmunotherapy will require large, well−controlled studies with appropriate comparators (55).

Mucin 1 (MUC1) was another tumor-associated antigen presented in pancreatic cancer cells, considering a promising antigen for immunotherapy (56, 57). The MUC1 peptide-loaded DC vaccine was elevated in patients after the surgical resection, which extended the medial survival time of patients to 26 months and exhibited favorable safety (58, 59). Pan et al. further modified the DC vaccines by loading the MUC1-PD-L1 immunogen (MUC1-Vax) that consist of a fusion gene that incorporated the extracellular domain of human MUC1 along with the programmed cell death ligand 1 (PD-L1), which demonstrated robust therapeutic effects against tumors in mice, indicating a promising strategy for treating pancreatic cancer by double-targeting MUC1 and PD-L1 (36). This innovation vaccine strategy should be further assessed in the clinical settings, further validating the safety and efficiencies in cancer patients. Similarly, Nagai et al. revealed that WT1/MUC1-DC vaccination extended the 3-year overall survival rate to 77.8% in the adjuvant setting of pancreatic cancer (37), which awaits to be assessed its clinical advantages in a large-scale trial. There are various approaches to load tumor antigens into DC vaccines, with earlier studies primarily focusing on the use of mRNA for this purpose (60). Utilizing circular RNA (circRNA) for antigen loading represents a promising alternative, as its inherent stability prolongs the duration of protein translation and enhances protein production compared to linear mRNA (61). Recent study utilized circRNA loaded FAPα and surviving(circRNAFS) and then transfected circRNAFS into DCs for vaccination (62). This vaccine combined with chemotherapy elicited significant immunogenic cell deaths and overcame immune evasion in Panc02 tumor model, which provided a novel insight into the clinical management of pancreatic cancer (62).

WTC vaccines represent a form of cancer immunotherapy that employs whole or lysed tumor cells, whether unaltered or modified, as a source of immunogenic components to elicit an anti-tumor response (63). Algenpantucel-L is an allogeneic pancreatic cancer vaccine composed of two human PDAC cell lines that express α-galactosyl (αGal) through retroviral transfer of the mouse αGT gene (38). This vaccine was evaluated as an adjunct to chemotherapy and radiotherapy in a study involving 70 patients who underwent surgical resection for PDAC and the inclusion of Algenpantucel-L resulted in improved disease-free survival (DFS) and overall survival (OS) (38). However, subsequent phase III trials did not validate the previous findings (39). To stimulate T-cell immune responses targeting different tumor antigens, researchers developed a pancreatic cancer vaccine known as GVAX, which was categorized as allogeneic and was composed of WTCs that secrete human GM-CSF (40, 64, 65). Furthermore, researchers revealed that the combination of GVAX, nivolumab, and urelumab (anti-CD137 agonist antibody) significantly elevated the levels of intratumoral cytotoxic T cells, demonstrating efficacy in PDAC patients undergoing neoadjuvant and adjuvant therapy (41). Recently, a clinical trial offered new insights into GVAX therapy, as the combination of the adjuvant GVAX vaccine and chemoradiotherapy showed good tolerance and moderate survival outcomes in PDAC patients, pending further validation in future studies (66). VIReST vaccine was developed by using pancreatic tumor cells derived from gene-edited induced pluripotent stem cells that were infected by viruses, which stimulated T cell immune responses, leading to a delay in malignant onset and progression in KPC transgenic mice (67), offering an innovation technological foundation for creating personalized cancer vaccines in high-risk population.

### 2.2 Nucleic acid-based vaccine

Nucleic acid-based cancer vaccines, encompassing both DNA and RNA formulations, signify a groundbreaking advancement in vaccination technology (68). These vaccines have garnered significant interest due to their accuracy, adaptability, and ease of production, positioning them as promising candidates for the development of personalized cancer vaccines (69). α-Enolase, known as ENO1, serves as an enzyme in the glycolytic pathway that was overexpressed in several cancers, including pancreatic cancer (70). Targeting ENO1 with monoclonal antibodies or silencing its expression could inhibit the migration of PDAC cells (71, 72). Researchers have developed ENO1 DNA vaccines that could prolong the survival of genetically engineered mice by eliciting cellular immune responses against ENO1 (73). However, the ENO1 DNA vaccine has no effect on the elimination of tumor; especially in the presence of phosphoinositide-3-kinaseγ (PI3Kγ) -mediated myeloid-derived suppressor cells (MDSC) recruitment within the TME (74, 75). Based on these findings, targeting MDSC through PI3Kγ inhibition in conjunction with ENO1 DNA vaccination could work synergistically to combat tumor growth in a B-cell-dependent immune response (76). Moreover, administering gemcitabine before ENO1 DNA vaccination activated significantly hindered tumor progression compared to mice that received either the vaccine or chemotherapy treatment alone, indicating the potential role of chemoimmunotherapy in pancreatic cancer therapy (77). Melanoma associated antigen A isoforms (MAGEA) played an essential role in modulating the interactions between tumor cells and stromal cells in PDAC (78, 79). Notably, vaccination with a MAGEA DNA vaccine targeting MAGEA2 and MAGEA10, could induce a robust immune response in chemotherapy-resistant mice (80). Administration of OsFS DNA vaccine that targeted human FAPα and survivin, significantly remodeling the immunosuppressive TME to impede tumor growth in PDAC (81). Furthermore, low-dose gemcitabine treatment could amplify the anti-tumor responses of OsFS, representing a promising approach for PDAC therapy (81). MUC1 DNA vaccines also exhibited potent anti-tumor effects on pancreatic cancer experimental models, which await further investigation in the clinical setting (82, 83). VVL−DD is a tumor−selective viral vaccine that demonstrated marked tumor selectivity *in vitro* and showed antitumor efficacy in a murine pancreatic cancer model *in vivo* (84).

Although advancements in DNA vaccines, however, DNA must first enter the nucleus, enabling extended production of target proteins, which arising a safe concern that integrating into the host genome (85, 86). Conversely, mRNA can be directly translated in the cytoplasm using the host cell’s machinery, which hold promise for developing cancer vaccine (87). The application of lipid nanoparticle (LNP) systems for mRNA vaccines, especially in the context of targeting personalized neoantigens, has emerged as a key area of interest (88, 89). Autogene cevumeran was an innovative mRNA vaccine that included RO7198457 and featured up to 20 patient-specific neoantigens intravenously delivered by LNPs (90), in combination with atezolizumab, and mFOLFIRINOX chemotherapy promoted significant T cell-mediated immune responses and linked to a delay in the recurrence of PDAC in the adjuvant setting (42). Follow-up results demonstrated that autogene cevumeran induced the generation of novel CD8^+^ T cells post-vaccination, which exhibited lasting effector functions and a memory-like T cell state for up to three years, potentially delaying the recurrence of PDAC (91). Currently, a global randomized trial (NCT05968326) is underway. Consequently, these results revealed that adjuvant mRNA-LNP neoantigen vaccines may address a significant challenge in pancreatic cancer vaccination.

### 2.3 Peptide-based vaccine

The peptide vaccine GV1001 consists of 16 amino acids sourced from the catalytic subunit of human telomerase reverse transcriptase (hTERT) (92). GV1001 elicited robust CD4^+^ and CD8^+^ T cell responses, as well as recognition by APCs (93). In earlier Phase I/II trials, T cell responses specific to GV1001 were observed in 50-80% of patients with PDAC who had an improved median survival (94). Notably, in patients with advanced PDAC who have high levels of eotaxin, the combination of GV1001 with chemotherapy resulted in improved OS and time to progression compared to patients treated with chemotherapy alone (43). A vaccine therapy employing three HLA-A2402-restricted peptides has shown promising effectiveness in clinical settings for treating patients with advanced PDAC (95). Recently, Cai et al. illustrated that targeting mesothelin (MSLN) could enhance the immune efficacy of neoantigen vaccines by diminishing cancer-associated fibroblasts (CAFs), which interrupted the conversion of naive CD4^+^ T cells into regulatory T cells, ultimately boosting t anti-tumor immunity (96). In line with this, VASH2-peptide vaccine, TM4SF5 peptide vaccine, and TGF-β- multipeptide vaccination inhibited PDAC progression in the preclinical studies (97–99), which await further validation in clinical trials. Moreover, ELI−002 2P vaccine employed Amph−modified mutant KRAS peptides (G12D, G12R; Amph−Peptides−2P) plus the CpG adjuvant Amph−CpG−7909 to optimize lymph−node delivery and heighten immunogenicity (100). In the phase 1 AMPLIFY-201 study, 84% of vaccinated pancreatic cancer patients mounted T-cell responses, tumor biomarkers declined in six individuals, and the median relapse-free survival (RFS) reached 16.33 months. T-cell reactivity predicted decreases in tumor biomarkers and ctDNA clearance, and was strongly associated with an 86% reduction in the risk of recurrence or death (101). ELI−002 exhibited potent therapeutic activity; however, further investigation is warranted.

## 3 Challenges in developing cancer vaccines

### 3.1 Low mutation burdens

Various factors related to the biology of PDAC that are unresponsive to immunotherapy, with hostile TME considered a key player in this process (102). This environment is highly immunosuppressive, marked by a low mutational burden and lack enough neoantigens (103). PDAC exhibit modern burdens in on-synonymous neo-antigenic mutations, leading to an absence of effective neoantigens (104). Neoantigens hold potential for enhancing personalized immunotherapy approaches for pancreatic cancer. A significant portion of neoantigens has been found to originate from atypical translation processes, which require further investigation to identify shared epitopes (105). Advancements in next-generation sequencing and developments in computational algorithms could uncover those antigens capable of effectively activating patients’ T cells, enabling their assessment in clinical studies, which may help overcome these limitations (106, 107). Moreover, immunosuppressive cells and factors present in the TME can hinder the anti-tumor immune response. Thus, developing effective cancer vaccines necessitates the implementation of strategies aimed at overcoming these immunosuppressive elements.

### 3.2 Technological obstacles in developed cancer vaccines

The technologies in developing pancreatic cancer vaccines are still in the early stages that pose multiple challenges. Compared to personalized neoantigen vaccines, public neoantigen vaccines can be manufactured in large quantities for immediate use, which reduces production time and lowers costs (108). However, a significant challenge is that the low number of shared neo-antigens among PDAC patients complicates the implementation of relevant treatment strategies, making them cumbersome and expensive (19, 109). To overcome the challenges, it is essential to implement standardized synthetic vaccine technologies that enable fully automated production and quality control, facilitating the establishment of efficient, miniaturized production lines (110). This approach enhances the overall efficiency and accessibility of vaccines for clinical applications in pancreatic cancer.

### 3.3 Lack of optimal preclinical models

Exploring the interactions and evaluating changes in immunological phenotype following anti-tumor treatments are considerably challenges due to the complexity and heterogeneity present in the TME of pancreatic tumors. Additionally, the weak immunogenicity of PDAC and its immunosuppressive characteristics complicate the development of animal models (111, 112). Genetically engineered preclinical models, created through gene modification and the introduction of mutations, represent the disease most accurately. However, these models cannot replicate the gradual accumulation of mutations seen in human pancreatic cancers, resulting in tumors that are relatively stable and less responsive to cancer vaccines (113, 114). These shortcomings highlight the necessity of integrating findings from animal models with clinical investigations to achieve a thorough understanding of immunotherapeutic approaches. Leveraging high-throughput methodologies to advance pancreatic cancer animal models will enable more faithful recapitulation of human disease features, thereby expediting the translation of preclinical discoveries into clinical practice.

### 3.4 Immunotherapeutic resistance mechanisms

The interplay between cancer and the immune system is intricate and dynamic. Adaptive immune resistance mechanisms enable tumors to protect themselves by adapting to immune attacks, even though they are recognized by the immune system (115). In PDAC driven by mutant KRAS (mKRAS), the downstream signaling pathways play a crucial role in immune evasion (116). For example, mKRAS facilitated the accumulation of MDSCs, CAFs and macrophages that inhibited cytotoxic T cell activity, as evidenced by autochthonous humanized mouse models of PDAC (117). Additionally, certain oncogenic pathways could induce T cell exclusion, allowing pancreatic tumors to evade immune attack. The activation of Wnt signaling pathway facilitated tumor development via upregulating expression of checkpoint molecules on T cells and inducing T cell exclusion, which contributed to immune escape (118). Furthermore, the loss of PTEN was linked to the activation of the PI3K-AKT signaling pathway and was associated with poor clinical responses to immunotherapy (119). While cancer vaccines could regulate TME, they often do not completely eliminate tumors when administered as standalone treatments due to these resistance mechanisms in PDAC (120, 121). Importantly, cancer vaccines can initiate epitope spreading, thereby expanding T cell responses and potentially improving the effectiveness of combination therapies (122). By leveraging the advantages of various treatment approaches, combination strategies can address the shortcomings of monotherapy and create a holistic method for targeting pancreatic tumors (123, 124).

## 4 Conclusions and perspectives

In recent decades, the recognitions on molecular mechanism of tumor cells evading immune detection have significantly improved, leading to notable advancements in cancer vaccines by mimicking natural immunity. With the advancements of sequencing technologies, the development of personalized cancer vaccines is likely to progress rapidly, which may revolutionize the therapeutic landscape of PDAC (125). Additionally, the careful selection of delivery systems is crucial for enhancing the immunotherapeutic responses (126). Recent technological advancements have introduced promising carriers, such as vaccines based on nanomaterials, which offer safer and durable immune responses (127). The integration of innovative strategies, meticulous candidate selection, and enhanced administration protocols has the potential to revolutionize cancer treatment, heralding a new era of therapeutic cancer vaccines. This progress ultimately paves the way for the effective use of cancer vaccines in the management of pancreatic cancer.
